# Acceptability of a shared cancer follow‐up model of care between general practitioners and radiation oncologists: A qualitative evaluation

**DOI:** 10.1111/hex.13846

**Published:** 2023-08-15

**Authors:** Tiffany Sandell, Heike Schütze, Andrew Miller

**Affiliations:** ^1^ School of Graduate Medicine Faculty of Science, Medicine and Health, University of Wollongong Wollongong New South Wales Australia; ^2^ Illawarra Shoalhaven Local Health District, Cancer Services Nowra New South Wales Australia; ^3^ Office of Medical Education Faculty of Medicine and Health, University of New South Wales Sydney New South Wales Australia

**Keywords:** cancer follow‐up, general practitioner, shared care, survivorship

## Abstract

**Introduction:**

Facilitators to implement shared cancer follow‐up care into clinical practice include mechanisms to allow the oncologist to continue overseeing the care of their patient, two‐way information sharing and clear follow‐up protocols for general practitioners (GPs). This paper aimed to evaluate patients, GPs and radiation oncologists (ROs) acceptance of a shared care intervention.

**Methods:**

Semi‐structured interviews were conducted pre‐ and post intervention with patients that were 3 years post radiotherapy treatment for breast, colorectal or prostate cancer, their RO, and their GP. Inductive and deductive thematical analysis was employed.

**Results:**

Thirty‐two participants were interviewed (19 patients, 9 GPs, and 4 ROs). Pre intervention, there was support for GPs to play a greater role in cancer follow‐up care, however, patients were concerned about the GPs cancer‐specific skills. Patients, GPs and ROs were concerned about increasing the GPs workload. Post intervention, participants were satisfied that the GPs had specific skills and that the impact on GP workload was comparable to writing a referral. However, GPs expressed concern about remuneration. GPs and ROs felt the model provided patient choice and were suitable for low‐risk, stable patients around 2–3 years post treatment. Patients emphasised that they trusted their RO to advise them on the most appropriate follow‐up model suited to their individual situation. The overall acceptance of shared care depended on successful health technology to connect the GP and RO. There were no differences in patient acceptance between rural, regional, and cancer types. ROs presented differences in acceptance for the different cancer types, with breast cancer strongly supported.

**Conclusion:**

Patients, GPs and ROs felt this shared cancer follow‐up model of care was acceptable, but only if the RO remained directly involved and the health technology worked. There is a need to review funding and advocate for health technology advances to support integration.

**Patient or Public Contribution:**

Patients treated with curative radiotherapy for breast, colorectal and prostate cancer, their RO and their GPs were actively involved in this study by giving their consent to be interviewed.

## 1 INTRODUCTION

Once cancer patients complete active treatment, they require long‐term follow‐up care to monitor for treatment‐related side effects and recurrence and provide psychosocial support.^1^, ^2^, ^3^ The oncologist‐led model is the most common and accepted follow‐up model of care for cancer survivors in Australia and internationally.^4^, ^5^, ^6^, ^7^, ^8^, ^9^, ^10^, ^11^, ^12^, ^13^ In this model, the oncologist manages patient care and side effects into the survivorship phase, often in a public or private hospital setting,^2^ and parallel to the care provided by the patient's general practitioner (GP). Although GPs are usually the first point of contact during a cancer diagnosis, GPs play a small or no role in cancer follow‐up care, and focus on other facets of the patient's health and well‐being.

The demand for cancer follow‐up care continues to increase globally.^14^ Subsequently, there is a need to explore and implement alternative models of cancer follow‐up care to address sustainability. While the oncologist‐led model suits many patients, it may not always meet patients' physical and psychosocial needs.^15^, ^16^ For some patients, a shared care model might be more appropriate, tailored to their tumour, treatments, locality (metropolitan, regional or rural), access to specialists, and their specific physical and emotional needs and preferences.^17^ A shared cancer follow‐up model of care is distinct from the sole transfer or discharge of care from one provider to another. A shared cancer follow‐up model of care harnesses the expertise of various health professionals^18^ and involves the explicit sharing of information and coordination of follow‐up care,^19^ usually between a GP and a specialist (for example their surgeon, medical oncologist, haematologist, or radiation oncologist [RO]). For shared care to be truly shared, the GP and the oncologist must be involved in each episode of care.

**Figure 1:**
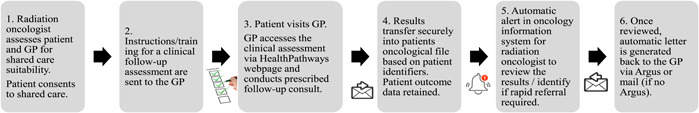
The radiation oncology shared follow‐up model of care process. GP, general practitioner.

Although shared cancer follow‐up care is safe in terms of quality of life and cancer recurrence,^20^, ^21^, ^22^, ^23^ barriers remain to implement it into clinical practice.^24^ There is a need for specific follow‐up guidelines for GPs^24^ and direct communication channels between the GP and the oncologist^25^, ^26^ to allow the oncologist to oversee their patient's care. Despite several shared care trials,^27^, ^28^ no mechanism has been utilised to facilitate the oncologist to remotely oversee care in real‐time. Secure health technology solutions to support shared cancer care remain unavailable.^29^ To address the above issues, we developed a shared cancer follow‐up model of care between GPs and ROs (see Figure 1). As part of the intervention, patients attended two cancer follow‐up appointments with their GP 6 months apart. This study aimed to evaluate the acceptability for this shared cancer follow‐up model of care among patients, GPs and ROs.

## 2 METHODS

### 2.1 Study design and setting

The study design was qualitative, with a critical realism lens. This intervention is part of a larger study described elsewhere.^30^ The study was conducted at two hospital cancer centres, one regional, Illawarra Cancer Care Centre, and one rural, Shoalhaven Cancer Care Centre, in New South Wales, Australia.

Australia's universal healthcare system, Medicare, provides free services in public hospitals and substantial coverage for other medical services for those who are eligible (citizens, permanent residents, reciprocal agreement visitors). Medicare provides rebates to patients for general practice consultations, however, may not always cover the full fee. Patients in this study did not have to pay at either the hospital cancer centre or their GP.

### 2.2 Participants and recruitment

ROs selected eligible patients from their follow‐up clinic list that were: scheduled for a radiation oncology follow‐up consultation in 2021; 3 years postradiotherapy treatment; breast, colorectal or prostate cancer; and suited to a shared cancer follow‐up model of care based on clinical considerations, including treatment type, prescription, and cancer staging. Patients were sent a participant information sheet outlining the purpose and process of the study and informed that participation was voluntary. Once a patient consented, their GP was invited to participate. All consent was received in writing. Data saturation usually occurs by around 12 interviews in qualitative studies^31^ therefore a target sample of 20 patients, their RO and GP was deemed sufficient.

### 2.3 Data collection

Semi‐structured interviews were conducted pre and post intervention. Interviews were initially conducted face‐to‐face at a private setting in a mutually agreed location and then changed to telephone due to COVID‐19 restrictions. Pre intervention, participants were asked about their views on the purpose of radiation oncology follow‐up care; they were then presented with the shared care model (see Figure 1) and asked their thoughts on the model. Post intervention (after patients attended two cancer follow‐up appointments with their GP 6 months apart), participants were asked about their experience of the shared care model. Interviews were audio‐recorded with consent (see Supporting Information: File S1 for a copy of the interview guide).

### 2.4 Data analysis

Interviews were transcribed verbatim with identifiable data removed and then imported into NVivo version 12^32^ for analysis. Using a critical realism lens, both inductive and deductive coding was employed, using the Theoretical Framework of Acceptability^33^ (TFA) (described further below) as a scaffold for the latter. To develop the coding framework, authors T. S. and H. S. coded one‐third of the data together and applied descriptive labels to sections of text, which were then organised into themes through an iterative inductive process. The process was then repeated to code the data deductively under the TFA domains. Following the development of the coding framework, T. S. coded the remaining interviews, which H. S. reviewed. Any discrepancies were discussed and resolved by consensus.

The TFA is a framework that facilitates the evaluation of health intervention acceptability based on the perceived and lived experience of the health intervention.^33^ The framework accounts for personal, organisational and other contextual factors that could influence the health intervention and acceptance. The TFA consists of seven constructs to determine overall acceptability (see Box 1).

BOX 1. **Theoretical framework of acceptability constructs^33^**

**Table:** 

TFA constructs	Definition
Affective attitude	How an individual feels about the intervention.
Burden	The perceived amount of effort that is required to participate in the intervention.
Ethicality	The extent to which the intervention has good fit with an individual's value system.
Intervention coherence	The extent to which the participant understands the intervention and how it works.
Opportunity costs	The extent to which benefits, profits, or values must be given up to engage in the intervention.
Perceived effectiveness	The extent to which the intervention is perceived to be likely to achieve its purpose.
Self‐efficacy	The participant's confidence that they can perform the behaviour(s) required to participate in the intervention.

A number of steps were taken to increase the rigour. To ensure the credibility of the data analysis, researcher triangulation was employed^34^ as described previously. Once data saturation was thought to have been reached, interviewing continued further to ensure that no new themes arose and that the varied views had been captured.^34^ Participants were not asked to validate the interpretations of their interviews (a process known as member checking or participant validation),^34^ out of respect for the amount of time that they had already afforded the study, and because doing so for the pre intervention interviews could have influenced the intervention. The COREQ‐32 checklist was used for reporting the findings.

### 2.5 Ethics approval and trial registration

Ethics approval was received from the Joint University of Wollongong and the Illawarra Shoalhaven Local Health District's Human Research Ethics Committee (2020/ETH00301). The trial was registered with the Australian New Zealand Clinical Trials Registry (ACTRN12620001083987).

## 3 RESULTS

Thirty‐two of the forty‐two participants from the overarching study were recruited for this portion of the study. This included 19 patients, 4 ROs, and 9 GPs. Participant demographics are shown in Tables 1 and 2. All patients and ROs contacted for interviews participated; GP reasons for not participating were due to time constraints. Sixty‐four (32 pre and 28 post intervention) interviews were conducted between February 2021 and February 2022. The average interview length was 16 min (range: 7–28 min).

**Table 1:** Patient demographics.

	*N*	%
Cancer		
Breast	10	53
Colorectal	1	5
Prostate	8	42
Total	19	100
Age		
50–59	2	10
60–69	10	53
70–79	4	21
80–89	3	16
Total	19	100
Sex		
Male	9	47
Female	10	53
Total	19	100
Location		
Shoalhaven Cancer Care Centre	7	37
Illawarra Cancer Care Centre	12	63
Total	19	100

**Table 2:** Provider demographics.

	General practitioner		Radiation oncologist		Total	
	*N*	%	*N*	%	*N*	%
Sex						
Male	5	56	–	–	–	–
Female	4	44	–	–	–	–
Total	9	100	4^a^	100	13	100
Age						
30–39	4	45	1	25	5	38.5
40–49	3	33	2	50	5	38.5
50–59	–	–	1	25	1	8
60+	2	22	–	–	2	15
Total	9	100	4	100	13	100
Training						
Australia	4	44	4	100	8	62
Overseas	5	56	–	–	5	38
Total	9	100	4	100	13	100
Years practicing (years)						
<10	3	33	2	50	5	38.5
11–20	4	45	1	25	5	38.5
>20	2	22	1	25	3	23
Total	9	100	4	100	13	100
Most recent oncology training (years)						
<10	3	34	4	100	7	55
10–20	2	22	–	–	2	15
>20	2	22	–	–	2	15
No training	2	22	–	–	2	15
Total	9	100	4	100	13	100

^a^ Due to the potential to identify participants' sex has not been itemised.

Five major themes were identified: Purpose of follow‐up care and views on the current oncologist‐led model; intervention coherence and affective attitude; burden and opportunity costs; ethicality and patient suitability; and self‐efficacy and perceived effectiveness. These are discussed below.

## 4 PURPOSE OF FOLLOW‐UP CARE AND VIEWS ON THE CURRENT ONCOLOGIST‐LED MODEL OF CARE

This theme relates to participants' views on the purpose of follow‐up care and their view of the current oncologist‐led model of care.

Participants felt the primary purpose of radiation oncology follow‐up care was to manage side effects, monitor for recurrence and to provide reassurance. In addition, ROs highlighted the importance of collecting clinical outcome data to report to the cancer registry and used for research:

*Pre intervention interview:* Of course, the last point about collecting clinical data and quality, the data somehow has to be kept in the cancer centre so that we can do quality projects and find out about the long‐term outcomes. (RO #2, rural)

Patients, GPs and ROs were positive about the oncologist‐led model, highlighting the benefit of easy access to multidisciplinary health professionals, imaging and other equipment:

*Pre intervention interview:* …if any other specialty needs to be involved or anything needs to be done procedure wise or anything, of course it's readily available. You can kind of do it probably in a better coordinated way because you're connected to the department. (GP #5, practicing >20 years, rural)

However, it was acknowledged that there was the possibility of duplication of care, and the oncologist‐led model may not suit everyone. While some patients felt more confident seeing a specialist for their follow‐up care, others, particularly those who were stable or lived far from the centre, felt it was inconvenient to go to the hospital just to be reassured that everything was okay.

Most patients believed their GP could play a role in their follow‐up care as they knew their long‐term health status and provided holistic care. However, few GPs stated that they help manage side effects, with most GPs stating they had a limited role in cancer follow‐up care, with referrals being their primary responsibility:

*Pre intervention interview:* Usually when they come to see me it about another health issue, and not specifically about the cancer, unless it is about a referral. (GP #9, practicing 11–20 years, regional)

Pre intervention, some patients assumed there was open communication and that information was shared between their GP and RO. Some patients were unaware that the Australian Government's *MyHealth Record* initiative did not give all health practitioners, including their doctors, access to their medical records.

## 5 INTERVENTION COHERENCE AND AFFECTIVE ATTITUDE

Intervention coherence and affective attitude related to participants understanding of what the intervention involved and how they felt about the intervention.

Patients, GPs and ROs recognised that shared care allowed ROs to focus on new patients, those on active treatment, and those with acute symptoms:

*Pre intervention interview:* I'm taking up quite a bit of time. I know it's only 15 minutes or something, but there's thousands of us, and I'll be quite happy to not use their precious time because they've got lots of other far sicker people that they're dealing with. (Patient #7, female, breast, age 60–69, rural)

ROs had a good understanding of the evidence to support GPs being involved in shared care and saw it as an opportunity to change their models of care without diminishing the quality of care. Although ROs were supportive, they acknowledged that it was important to ensure that the GP was comfortable and felt adequately supported:

*Post intervention interview:* I suppose the only thing would be making sure that the GP on their end is comfortable with that and making sure that they feel that they're supported enough to take on this role. (RO #4, rural)

Although there was good support for the GP to play a greater role in cancer follow‐up care, some patients in the pre intervention interviews questioned their GP's cancer‐specific skills. However, both GPs and ROs felt confident in the GP's ability to participate in this shared care model:

*Pre intervention interview:* They'll be assessing for side effects, which usually is very minimal, and the side effects haven't really changed that much in the last 20 years [for breast cancer]…therefore I think using primary care physicians to help with the follow‐up, I think it's a good idea. (RO #2, rural)

The relationships and continuity of care that existed between patients and their doctors also influenced how participants felt about this shared care model. Patients with a good and longstanding relationship with their GP trusted them and felt positive about the model:

*Pre intervention interview:* Just the fact he's been with us for 30 years means that he sort of actually knows how we think, he knows what we've been through…so I trust [my GP] emphatically. (Patient #8, male, prostate, age 60–69, regional)

Some patients acknowledged the need to train registrars and balance the demands of hospital resources, however, patients who had little continuity of care with their RO felt more positively about this shared care model:

*Pre intervention interview*: …there's a certain detachment. I only ever saw [RO] once… I haven't seen the same person twice…But going in each time and getting someone different is a bit daunting whereas having [my GP] is not. (Patient #6, female, breast, age 70–79, regional)

After experiencing the shared care model, one patient that preferred the oncologist‐led model acknowledged their preference was based on the relationship with their RO:

*Post intervention interview:* I guess if I wasn't so happy with [my RO], it may lead me more towards the GP stuff, but because it is so well organised and run in there, I actually don't mind going in there. (Patient #19, male, prostate, age 60–69, regional)

## 6 BURDEN AND OPPORTUNITY COSTS

This theme relates to the amount of effort and any sacrifices required to participate in this shared care model.

Pre‐intervention, there were mixed views about the extra time demands this shared care model would place on GPs. Patients did not want to add extra work to their GP, GPs were uncertain how much it would impact their workload, and ROs acknowledged this model could be a time burden for GPs:

*Pre intervention interview:* I would love it. I think that it's sorely needed but it potentially might be a source of an added burden to their [the GP] already existing schedule, which is full. (RO #1, regional)

Post intervention, GPs felt the consultation was similar to a standard clinical review for other chronic health conditions such as diabetes or cardiovascular care. Some GPs stated it took longer than a standard appointment and noted that they spend different amounts of time with patients based on their medical needs. However, most GPs stated that there was no notable time burden, and some likened the time as being similar to writing a referral:

*Post intervention interview:* It's no big deal. It's actually probably easier than writing a referral. (GP #4, practicing >20 years, regional)

Although the model did not notably increase workload, there were concerns that future work may increase depending on the complexity and number of patients:

*Post intervention interview:* If it is just for prostate cancer or for a stable patient, I guess it wouldn't be too sort of time consuming. But if it then started to include breast cancer patients, I think that would probably end up taking a bit more time. (GP #8, practicing 11–20 years, rural)

ROs felt that this model did not increase their time burden as they remotely monitored the patient's clinical assessment. One RO highlighted how the automatically generated letter back to the GP upon their review saved their time and additional administrative staff time. However, ROs felt it was too soon to identify if the model would make a difference in the long term:

*Post intervention interview:* There hasn't been too many patients, but from the few that have gone to their GPs, I feel good about it. I think it worked well. It is not enough to make a dent in my clinic schedule yet though. (RO #3, regional)

Participants felt that the model alleviated some burdens on patients, especially physical access to care. Patients appreciated greater flexibility with rescheduling appointments and not waiting months for a new appointment. Patients appreciated not having to travel the distance to the hospital for their follow‐up appointment when their GP was closer:

*Post intervention interview:* …to be able to go to the local [my GP] is only 10 minutes away…Whereas the [RO], obviously, a day trip, which I don't mind… It's not a killer of a thing, but there are times when you don't have the time. (Patient #1, female, breast, age 70–79, rural)

Patients who had GPs that bulk billed (i.e., no copayment was required), felt that outside of this intervention, cost was not a burden to this model. However, patients who were required to make a copayment to visit their GP, identified cost as a barrier in the future:

*Post intervention interview:* [The GP is] actually charging $30…And I've got to go and see him monthly now because of [other health issues]. So, I've got to see him at least once a month now and then adding that extra. (Patient #12, female, breast, age 50–59, regional)

Some GPs highlighted that in order for the model to work, there would need to be appropriate government funding to remunerate it on a time‐based consultation:

*Post intervention interview:* There would need to be an [Medicare] item code for this. Even though I'm able to do it there needs to be better support if I'm to actually do this. (GP #3, practicing 11–20 years, regional)

## 7 ETHICALITY AND PATIENT SUITABILITY

Ethicality explored the participant's view of this shared care model regarding its appropriateness with their personal and professional value system, including patient suitability for shared care.

GPs and ROs acknowledged that this shared care model might not suit every patient and recognised that some patients might want to continue with the oncologist‐led model if they were anxious or preferred the RO:

*Pre intervention interview:* I have some patients that enjoy going to the specialist, just to have to big power say they are doing ok. (GP #9, practicing 11–20 years, regional)

Although GPs and ROs saw the model as providing a choice to the patient, patients trusted their RO, and wanted their RO to advise them on the most appropriate follow‐up model suited to their individual situation:

*Pre intervention interview:* Trust is the major feature. Distance and cost is secondary. (Patient #14, male, prostate, age 80–89, regional)

*Post intervention interview:* It's whatever the professionals think is best. Because they have my best interests at heart. (Patient #6, female, breast, age 70–79, regional)

GPs and ROs recognised that patients needed to be carefully selected based on their post treatment stage. Transitioning away from the oncologist‐led model to shared care would be best 2–3 years post treatment in stable patients:

*Post intervention interview:* Especially in the initial first two years, it's really important to get the hospital fully involved and making sure everything is good. But it is the future years, when we're moving on beyond the two‐year mark, then that is the time when the patients really don't want to go to the hospital. They want that sort of care to be available at the GP. (GP #3, practicing 11–20 years, regional)

However, ROs had varied views on which cancers were suitable for this model of care. The implementation of funded telehealth during the COVID‐19 pandemic influenced some ROs views on prostate cancer. Some ROs felt that telehealth follow‐up for prostate patients was appropriate as it removed some access issues for patients. All ROs thought breast cancer patients would be suitable for this shared care model based on improved treatments, known side effects and the need for a physical examination and review of the skin:

*Post intervention interview:* Telehealth has its advantages. But shared care, well if the patient is still physically seen by the GP, to assess their skin, that is important. (RO #2, rural)

Although GPs' and ROs' views aligned on having stable patients in this shared care model, some GPs and ROs highlighted concerns about liability and who maintained overall responsibility for the patient:

*Post intervention interview:* Who takes the responsibility?…So if something was to happen, it's not really my responsibility because the information that I have is limited…So I think that there is a remote monitoring, but I think ultimately, and I'm just saying that if something were to go wrong, the GP missed a recurrence, it's still the GP's responsibility, or his responsibility to have raised a concern. (RO #2, rural)

## 8 SELF‐EFFICACY AND PERCEIVED EFFECTIVENESS

This theme related to whether the participants felt that they could carry out what was required of them to participate in shared care, and their views on the effectiveness of this shared care intervention.

Post intervention, ROs maintained their positive view and reported that their patients appreciated their GP being more involved in their care, and that it allowed them to know more about their follow‐up care. Many patients highlighted that the follow‐up review with their GP was the same as if they had seen their RO:

*Post intervention interview:* She gave me a physical examination sitting up and lying down and everything was basically exactly the same as what happens when I've gone in the past to the cancer centre. (Patient #7, female, breast, age 60–69, rural)

Post intervention, all GPs felt confident in performing the follow‐up review, and one highlighted that their confidence in had improved:

*Post intervention interview:* I was really apprehensive to start with, but it was really good. I mean, it went very well and I could do it. (GP #3, practicing 11–20 years, regional)

Providing a clinical assessment protocol for the GP was important in allowing the GP to know specifically what information the RO wanted. Being able to access it from a frequently used GP website (HealthPathways) was viewed positively. The use of the protocol was also identified as a self‐learning tool for GPs:

*Post intervention interview:* Having a framework that says this, this, and this, is really good because it makes sure we don't miss anything. And then, obviously for your viewpoint, that gives you consistency in the feedback that you are getting. So yeah, I've found that all very, very good. (GP #4, practicing >20 years, regional)

Patients, GPs and ROs felt confident the patient would be referred quickly back to the RO if any recurrence was suspected via the rapid referral option. Although the rapid referral option in the clinical assessment protocol was not used within this study, participants likened the intervention mechanism to a safety net:

*Post intervention interview:* It's the convenience to the patient and also the safety net. (GP #5, practicing >20 years, rural)

An important factor in the acceptance of this model depended on the electronic communication system working properly so that the GP could access the clinical assessment protocol and that the transfer of information to the RO worked in real‐time:

*Post intervention interview:* The electronic media system is certainly making this… When it works, these sort of situations are very streamlined, but of course when they don't work though, totally wretched. (Patient #1, female, breast, age 70–79, rural)

While some GPs had electronic recall scheduling systems for their patients, not all did, and this was viewed as essential for a shared care model:

*Post intervention interview:* I think main thing is the time and also the recall system. We have a robust recall but for different reasons, but not for this cancer follow‐up… so that's not included in our software. (GP #1, practicing 11–20 years, regional)

The fact that the RO maintained involvement was important for patients' acceptance. Patients wanted the RO to stay involved. Some patients also felt it was important to know if their RO had reviewed the clinical assessment results:

*Post intervention interview:* I guess I don't know if [my RO] saw anything. Well I guess just me needing to know that [my RO] is actually still involved. (Patient #3, female, breast, age 50–59, rural)

The ROs felt comfortable with the transfer of information from the GP to the hospital system. They also thought the clinical information received was consistent with what they observed from their clinical practice. However, there remain further requirements to improve overall acceptance:

*Post intervention interview:* It's a good concept. I still think there might need to be some more work to individualise the clinical assessments, I know they reflect our consultations, but sometimes we might get patients that need that unique item reviewed. (RO #3, regional)

GPs and ROs acknowledged there was a need to manage patient expectations and that if the patient expects their follow‐up care to be with the RO they may be disappointed if their follow‐up care is with the GP. ROs recognised they played an important role in advocating the shared care model to their patients. Patients and GPs viewed the shared care model positively. ROs saw this model as important to continue to provide safe follow‐up care to suitable patients in general practice, whilst improving access for newly diagnosed patients and those with recurrence:

*Post intervention interview:* So I'm satisfied that it's feasible and practical…I think now that I've moved from being one who's supportive to one who feels that it's absolutely necessary in order for us to maintain services for newer patients who come through because otherwise we'll be overwhelmed. (RO#1, regional)

## 9 DISCUSSION

Concerns about the sustainability of the current oncologist‐led model for cancer follow‐up care have led to the need to explore alternative models of care.^27^, ^28^ Despite studies showing acceptance for shared cancer follow‐up care,^35^, ^36^ barriers remain to implement the model into clinical practice.^24^, ^37^, ^38^ A unique feature of the intervention evaluated in this study was the direct use of health technology to bridge the communication gap between GPs and ROs and specific protocols to support shared cancer follow‐up care.

A documented barrier to shared cancer follow‐up care is that patients and oncologists perceived that GPs lacked the cancer‐specific skills required.^38^, ^39^, ^40^ Similarly, our study found that patients had the same concerns pre intervention. However, post intervention patients felt confident that their GPs had the requisite skills and found it comparable to an RO follow‐up assessment. The ROs in our study did not have concerns about the GPs ability to perform follow‐up using the prescribed clinical assessment protocol.

Clear protocols are thought to foster acceptance for shared cancer care.^25^, ^41^, ^42^ We provided a specific clinical assessment protocol for GPs to follow. GPs appreciated knowing what information the RO needed for follow‐up care, felt it acted as a self‐learning tool, and felt the protocol was easy to follow and to integrate into their work. ROs saw that the GPs' assessment results were similar to their assessment results, increasing their acceptability of the model. However, ROs saw an opportunity to further individualise patients' clinical assessments, noting that patients have had different treatments with varying side effects and toxicities to monitor.

Another concern about adopting shared care was the additional burdens the model would place on GPs.^43^, ^44^, ^45^, ^46^ Although this was an initial concern from the participants, our post intervention results showed that GPs found the time taken to assess the patient in this model to be no more burdensome than writing a referral. However, GPs had reservations about what types of cancers and how many patients would be expected to transition from the oncologist‐led model to the shared care model in the future, and how that would impact their workload.

GPs and ROs felt that transitioning from the oncologist‐led model to the shared care model would be best for low‐risk and stable patients, consistent with a recent Australian qualitative study.^47^ Additionally, GPs and ROs thought the transition would be appropriate for patients 2–3 years post treatment. Some ROs felt that the recent changes to telehealth funding due to the COVID‐19 pandemic made telehealth follow‐up for prostate patients preferable to this shared care model. However, ROs saw the benefit of this shared care model for breast patients, as they required a physical examination and review of their skin.

GPs and ROs saw the benefits for this shared care model for patients in rural areas. Interestingly, shared care has been suggested as a way to improve access for patients in rural areas^48^; while this may be true, access was not emphasised as a benefit by rural patients in this study to any greater extent than for regional patients. Nevertheless, the adoption of a shared care model is about patient choice.

GPs and ROs felt that it was the patient's choice to participate in shared care. Patient choice aligns with person‐centred care principles.^49^ However, patients emphasised that they trust their RO to advise them on the most appropriate follow‐up model suited to their individual situation. This trust is the foundation of the doctor–patient relationship, and patients who have that trust are more likely to adhere to the plan recommended to them.^50^ GPs also looked to the RO for support and guidance to foster the adoption of a shared care model. It has been reported that relationships were poor between GPs and oncologists,^51^ and the ROs in this study recognised that they have an opportunity to increase their role in supporting GPs in the transition to a shared care model.

This shared care model relied heavily on health technology in both the GP and RO settings. The use of health technology to better connect the GP and RO acted as a safety net for participants in this shared care model. GPs and patients valued rapid access to oncologist assessment for new symptoms, or for suspected recurrence. Providing the clinical assessment online via HealthPathways and transferring the clinical information directly into the hospital's medical record system allowed the RO to oversee care and fostered acceptance for this shared model. For ROs, the retention and access to patient outcome data were integral to acceptance, which is seldom discussed in other studies.^52^ ROs within this study were very clear that any model moving forward needed to integrate with the hospital's system so that they could continue to use the clinical outcome data of their patients for their medical reviews, reporting requirements to state or national bodies, and use in research. Additionally, existing electronic medical record systems routinely used in general practice use recalls systems, and while they may not exist for cancer follow‐up, they can be customised to allow recall.

Although this study has attempted to overcome some barriers to adopting shared care, this study has highlighted an additional barrier, remuneration to the GP and cost to the patient. An Australian study found that a shared cancer follow‐up model is cheaper than usual care.^27^ Promoting the cost‐effectiveness of shared care is important for advocating changes to models of care and funding at a federal level. However, consideration is also required to understand the cost impact of shared care on patients. In this study, there are no patient copayments for RO consultations in the public hospital system, and although Medicare can cover GP consultation fees, many patients may be charged a copayment.^53^ Although patients made no copayment to their GP for this study, this was highlighted as a barrier by patients who have GPs that charge copayments. Consequently, adopting a shared cancer follow‐up model may impose additional costs on patients to visit their GP.

There is a need to review the funding model and its implications for the patient, GP and RO. There is a need to determine an appropriate Medicare item that addresses the GP's remuneration needs on a time‐based consultation and minimise the need to charge patients a copayment. Additionally, in Australia, hospitals are funded based on their activity; therefore, by reducing the RO's hospital activity, the model could potentially reduce the funding provided to the facility and to the ROs for research and development purposes. However, considering that ROs will still be directly involved in remotely monitoring the patient's medical record, this activity should be captured and remunerated.

### 9.1 Strengths and limitations

This study has several strengths. Firstly, its varied sample of participants provided the ability to examine different perspectives of this shared cancer follow‐up model of care. This meant that their views of follow‐up cancer care and shared care were tangible and considered the interpersonal dynamics of the empirical, actual and real relationships. Secondly, the use of health technology as the mechanism to support a streamlined shared care model was integral to the success of this model of care. Finally, frameworks are often underutilised in health implementation studies^54^; using the TFA contributes to the field as a whole and the topic.

This study has some limitations. During data collection interviews changed from face‐to‐face to telephone interviews due to the COVID‐19 travel restrictions. While some consider face‐to‐face interviews as the gold standard,^55^ current research has not found variations in data quality.^56^, ^57^ This study was in a rural and regional Australian setting with publicly funded ROs, and the results may not apply to metropolitan, private or international settings. Although data saturation was obtained, the smaller sample size of GPs and ROs is acknowledged as a potential limitation. This study was limited to the radiation oncology setting, and did not include surgical, medical oncology or haematology disciplines, and the results may not be transferable. Discipline‐specific trials are recommended.

## 10 CONCLUSION

This radiation oncology‐shared cancer follow‐up model of care is acceptable to the patients, GPs and ROs. Central to the acceptance was the health technology used within the intervention: the clinical assessment protocol accessible via a website and the system that transferred the results securely from the GP to the RO in real‐time, allowing the RO to maintain involvement, oversee care and collect outcome data. The mechanisms acted as a safety net for patients, GPs and the RO. To effectively adopt this radiation oncology‐shared cancer follow‐up model of care in clinical practice, addressing the implications for practice and policy is essential. This necessitates collaboration with GPs and ROs to increase their confidence that GPs have the requisite skills for shared care and for the model to be endorsed and supported by ROs. There is a need to review funding to support health technology integration that underpins this model, while prioritising appropriate remuneration for both GPs and ROs for their time and expertise. Further research and analysis to explore this model's long‐term benefits and sustainability in clinical practice is recommended.

## AUTHOR CONTRIBUTIONS

T.S. conceptualised the model of care intervention with support and advice from A.M. A.M. designed the electronic interface. T.S, H.S. and A.M. designed the study. T.S. and H.S. were responsible for ethics. T.S. collected the data. T.S. and H.S. conducted the data analysis. T.S. drafted the original manuscript. H.S. substantially revised following drafts and A.M. reviewed all drafts. All authors have read and agreed to the published version of the manuscript.

## CONFLICT OF INTEREST STATEMENT

The authors declare no conflict of interest.

## ETHICS STATEMENT

This study was performed in line with the principles of the Declaration of Helsinki. Approval was granted by the Joint University of Wollongong and Illawarra Shoalhaven Local Health District's Human Research Ethics Committee, 2020/ETH00301. Informed consent was obtained from all individual participants included in the study. The authors affirm that human research participants provided informed consent for publication.

## Supporting information

Supporting information.

## Data Availability

The datasets generated during and analysed during the current study are available from the corresponding author on reasonable request.
