# Supplementary material for: Acceptability of a shared cancer follow‐up model of care between general practitioners and radiation oncologists: A qualitative evaluation
Source: Health Expect. 2023 Aug 15;26(6):2441–52. doi: 10.1111/hex.13846 (PMC10632636; doi:10.1111/hex.13846)
Supplement: Supplementary file 1 — Supporting information. [file HEX-26--s001.docx]

Supplementary File 1. Semi-structured interview guide.

| 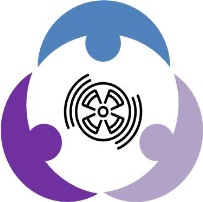 | **Semi-structured interview guide:** |
| --- | --- |

**PRE IMPLEMENTATION INTERVIEW GUIDE**

**Introductory script**

- Thank participant for agreeing to take part in this research.
- Introduce the research and briefly discuss its purpose.
- Discuss the role of the interviewer: to raise topics for discussion and then to listen as the participant shares their views and experiences.
- Reassure the participant that they are free to talk about any aspect of their experience or attitudes. There are no right or wrong or even typical answers to any of the questions that we will discuss.
- Remind the participant that, with their permission, the interview will be audio-taped.
- Reassure confidentiality and the participant’s right to stop the interview at any time.
- Clarify that the interview will take approximately 30 minutes.
- Ask whether the participant has any questions before beginning.

**General practitioner/Radiation Oncologist**

**1. Role of cancer follow-up/perceived goals**

- How would you describe the concept behind radiation oncology cancer follow up?
- In your view, what are the aims of radiation oncology cancer follow up?
- In your view, what do patients want from radiation oncology cancer (radiation oncology) follow up?

**2. Experience of current follow up strategy**

- Please describe your current role, *(for GPs if any)*, in the cancer follow up care of (Patient X)
  - How do you feel about your current role?
  - Are there any aspects of this role that you feel uncomfortable with?
  - Would you like to change anything about this role/your input?
  - What aspects of follow up care, if any, do you feel responsible for?

**3. Views on hospital, specialist-led follow up**

- What are views on the current hospital-led cancer follow up model for this patient?
- Are there any particular strengths of the hospital-led follow-up?
- Are there any problems with hospital-led follow up?
- Would you change anything about the current follow up?
- What impact does cancer follow up have on your clinical workload?
- Do you experience any challenges when providing cancer follow-up?

**4. Follow up provider**

- Who do you believe is best placed to provide cancer follow up?
  - Why do you think this provider is best placed?
  - Are there any situations where another provider would be best places to provide follow up?
  - Are there any patient factors that in your mind would make someone require a different follow-up provider?

**5. Views on primary care led follow-up**

Participants are presented with the shared-care model.

- How would you feel about a GP taking a greater and/or sharing the role in cancer (radiation oncology) follow-up?
- What would you see as the potential advantages in this model?
- What would you see as the potential disadvantages in this model?

(Think about issues such as costs, convenience, confidence in treating cancer patients amongst the health care professionals, ability for the follow up to detect important recurrences etc.)

- In your view, what would patients think about this proposed shared-care model?

**6. Acceptability of participating in shared-care**

- Finally the general practitioners/radiation oncologists will be asked about their views on the proposed shared-care model and if they could choose between the current model, or the shared-care model, what would they choose.

**7. Conclude Interview**

- As this is a new area of research, we may have missed something important in this interview. Is there anything else that you would like to comment on?

**Interview debriefing and closure**

- Thank the participant for sharing their views.

**Patient**

**Additional introductory script to patients:**

- Reassure that their specialist and general practitioner will not have access to what the patient says in the interview – everything will be completely confidential.
- Clarify that the interview will take approximately 30 minutes.

1. **Role of cancer follow up/perceived goals**

- What do you believe to be the purpose of follow-up appointments following radiotherapy treatment for your cancer?
- What do expect from your cancer follow-up?
  - Have you discussed your follow-up plan with a doctor or nurse?

**2. Experience of current follow up strategy**

- Please can you describe the current follow-up care you are receiving?
  - Where are you receiving this follow-up?
  - What individuals or groups are providing this follow-up?
  - How frequent are these appointments?

1. **Views on hospital, specialist-led follow up**

- What do you think of the current follow-up plan?
- Does this current plan meet your expectations of follow-up?
- Are there any particular strengths of your current follow-up?
- Are there any problems with your current follow-up?
- Would you change anything about your current follow-up?
- What role does your GP currently play in your cancer follow-up?

**4. Follow up provider**

- Who do you believe is best placed to provide this follow-up?
  - Why do you think this provider/these providers is/are best placed?
  - Are there any situations where this may change and the hospital/GP may be best placed to provide follow up?
  - In which situations is the hospital/specialist best placed to provide follow-up
  - In which situations is your GP best placed to provide follow-up.

**5. Views on primary care led follow up**

Participants would then be presented with the model of care highlighting the radiation oncologist is overseeing the care in real-time.

- How would you feel about your GP taking a greater role and sharing in your cancer follow up?
- What would you see as the potential advantages in this approach?
- What would you see as the potential disadvantages in this approach?

(Think about issues such as costs, convenience, time, confidence in health care professionals, ability for the follow up to detect important recurrences etc.)

**6. Acceptability of shared-care model**

- If they knew their radiation oncologist could oversee their care, in real-time (with the transfer of medical information), would they choose usual care, or shared-care.

**7. Conclude Interview**

- As this is a new area of research, we may have missed something important in this interview. Is there anything else that you would like to comment on?

**Interview debriefing and closure**

- Thank the participant for sharing their experiences.

**POST-IMPLEMENTATION SEMI-STRUCTURED INTERVIEW GUIDE**

**Introductory script**

- Thank participant for taking part in this research.
- Briefly reiterate its purpose.
- Clarify that the interview will be shorter than the first, around 20 minutes.
- Ask whether the participant has any questions before we begin.

**General practitioner/Radiation Oncologist**

**1. Experience of shared-care follow-up strategy**

- Please can you describe your experience of shared-care for (Patient X)
  - How do you feel about your shared-care role?
  - Are there any aspects of this shared-care role that you felt uncomfortable with?
  - Would you like to change anything about this role/your input?
  - Now that you have been involved with shared-care, what aspects of follow-up care, if any, do you feel responsible for?

**2. Views on shared-care**

- Are there any particular strengths/advantages of shared-care follow-up?
- Are there any problems/disadvantages with shared-care follow-up?
- Would you change anything about the shared-care follow-up?
- What impact did shared-care follow-up have on your clinical workload?
- Did you experience any challenges when providing shared-care follow-up?
- In your view, what would patients think about their experience with this shared-care model?

**3. Acceptability of participating in shared-care**

- Finally the general practitioners/radiation oncologists will be asked if they could choose between the current model, or the shared-care model, what would they choose.

**4. Conclude Interview**

- As this is a new area of research, we may have missed something important in this interview. Is there anything else that you would like to comment on?

**Interview debriefing and closure**

- Thank the participant for sharing their views.

**Patient**

**Additional introductory script to patients:**

- Reassure that their specialist and general practitioner will not have access to what patient says in interview – everything will be completely confidential.
- Clarify that the interview will be shorter than the first, approximately 30 minutes.

**1. Experience of shared-care follow-up strategy**

- Please can you describe your experience of shared-care between your GP and radiation oncologist.
  - How do you feel about shared-care?
  - Are there any aspects of shared-care that you felt uncomfortable with?
- Are there any particular strengths of shared-care follow-up?
- Are there any problems with shared-care follow-up?
- Would you change anything about the shared-care follow-up?
- What impact did shared-care follow-up have on your daily life?
- How did you feel knowing your radiation oncologist could see the results of your follow-up appointment immediately?

**3. Acceptability of participating in shared-care**

- If they could choose between the current hospital-based model, or the shared-care model, what would they choose.

**4. Conclude Interview**

- As this is a new area of research, we may have missed something important in this interview. Is there anything else that you would like to comment on?

**Interview debriefing and closure**

- Thank the participant for sharing their views.
